# Dissociated prismatic loop punching by bubble growth in FCC metals

**DOI:** 10.1038/s41598-021-92219-7

**Published:** 2021-06-18

**Authors:** Miaomiao Jin, Yipeng Gao, Yongfeng Zhang, Chao Jiang, Jian Gan

**Affiliations:** 1 Department of Nuclear Engineering, The Pennsylvania State University, 205 Hallowell Bldg., University Park, PA 16802 USA; 2 Idaho National Laboratory, 2525 Fremont Ave, Idaho Falls, ID 83402 USA; 3 Department of Engineering Physics, University of Wisconsin, 1500 Engineering Drive, Madison, WI 53706 USA

**Keywords:** Metals and alloys, Mechanical engineering

## Abstract

Materials performance can be significantly degraded due to bubble generation. In this work, the bubble growth process is elaborated in Cu by atomistic modeling to bridge the gap of experimental observations. Upon continuous He implantation, bubble growth is accommodated first by nucleation of dislocation network from bubble surface, then formation of dissociated prismatic dislocation loop (DPDL), and final DPDL emission in $\langle 110\rangle$ directions. As the DPDL is found capable of collecting He atoms, this process is likely to assist the formation of self-organized bubble superlattice, which has been reported from experiments. Moreover, the pressurized bubble in solid state manifests the shape of an imperfect octahedron, built by Cu $\{111\}$ surfaces, consistent with experiments. These atomistic details integrating experimental work fill the gap of mechanistic understanding of athermal bubble growth in Cu. Importantly, by associating with nanoindentation testings, DPDL punching by bubble growth arguably applies to various FCC (face-centered cubic) metals such as Au, Ag, Ni, and Al.

## Introduction

He bubble nucleation and growth are of significance to evaluate materials performance under irradiation, including dimensional stability and mechanical properties of materials systems such as the structural materials in fission and fusion reactors^1^. They involve an interplay between the clustering of He atoms with high binding energy and low solubility, and the response of matrix materials. A fundamental understanding of bubble-matrix interaction during bubble growth is critical to complement the understanding of macroscale material degradation, and constitutes a prerequisite of high-fidelity continuum models for predicative modeling.

He bubbles have received active interest from both experiments and simulations due to the inevitable existence of He in both fission and fusion systems. Experimentally, He atoms are commonly utilized as incident ions or co-implanted with other heavy ions to examine He evolution and radiation effects in materials such as embitterment; bubbles are frequently observed under in-/ex-situ characterization techniques^1^. Going beyond the resolution limit of experimental techniques, bubble growth can be nicely captured by molecular dynamics (MD) with atomistic details, yielding multiple studies on the bubble growth mechanisms in BCC metals such as W and Fe. Overall, bubble growth can occur via two modes: (1) diffusion of vacancy-He complex, or even bubble itself^1,2^; and (2) aggregation of He interstitials. The former, due to the rate-limiting step of diffusion with high migration barriers, is only active at high temperatures. At low temperatures, the latter becomes dominant, which can be effectively examined via MD simulations by controlling bubble volume and He content. As bubble becomes over-pressurized, the growth is accommodated by punching out matrix atoms in several forms: individual interstitials^3,4^, multiple interstitials altogether which rearrange into a prismatic dislocation loop^5–8^, or single dislocations which evolve into a prismatic dislocation loop^9^. These punched out entities are emitted from the bubble, releasing the high local strain caused by bubble growth. Early theoretical analyses based on energetics were carried out to identify the punching criteria^10,11^.

Compared to abundant mechanistic examinations on BCC metals aforementioned, bubble growth in FCC metals has much less attention, despite bubbles have also been frequently reported^1,12–14^. It is perceived, however, that FCC metals may share similarities to BCC metals. For example, Wolfer theoretically analyzed dislocation loop punching by a single bubble for Ni at room temperature^11^. Hetherly et al. touched upon loop punching in the context of preferential bubble nucleation at twist grain boundary in Cu and Au^12^. Although investigations on He migration and clustering, and bubble migration and coalescence in FCC metals such as Ni^15,16^, Al^13^, and Cu^12,14^ were conducted, the detailed bubble growth process, especially a fundamental understanding on the punching mechanism is still at lack. Hence, in this work, we aim to elaborate on He bubble growth in Cu–He system using MD simulations, and report a novel atomistic process involving incipient plasticity of punching dissociated prismatic dislocation loop (DPDL) during bubble growth, which then assists microstructure evolution. Inferring from nanoindention testings which share similar characteristics to the current work, it is believed that this finding also applies to other FCC elemental metals such as Au, Ag, Ni, and Al.

## Results

**Figure 1:**
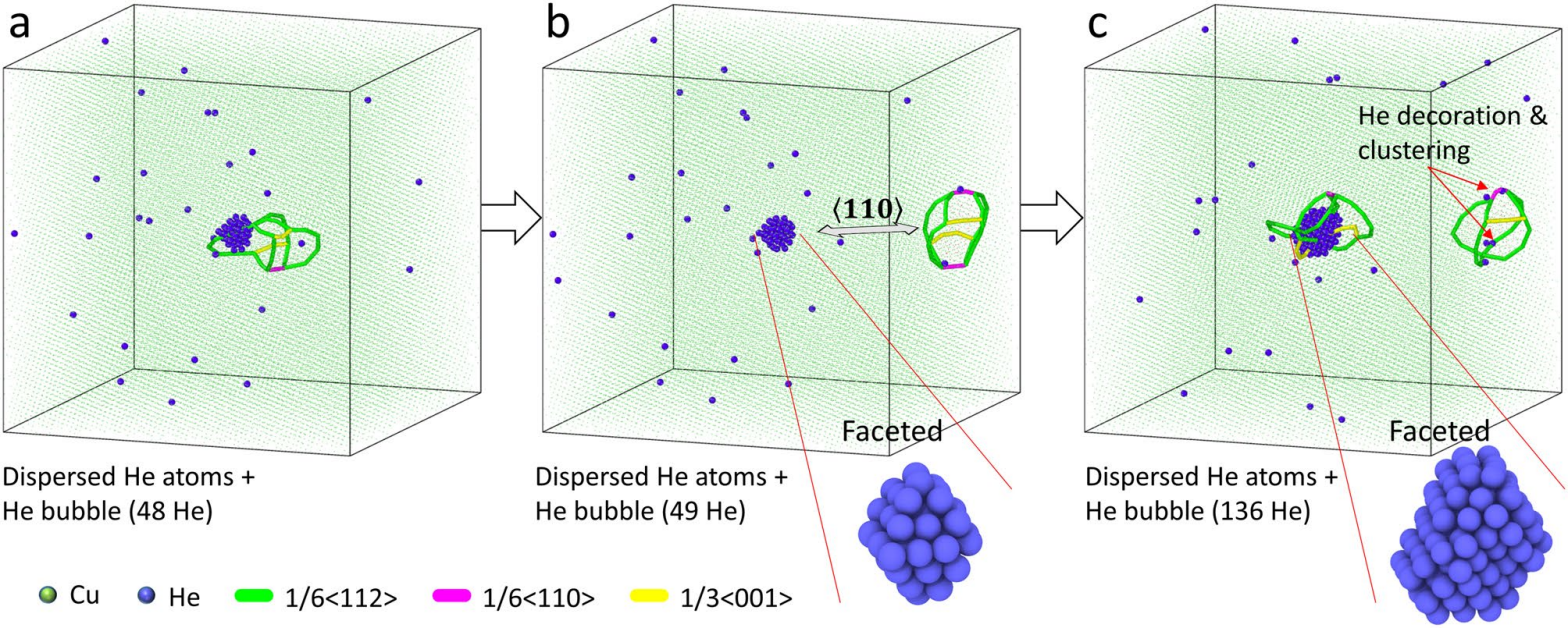
DPDL punching during the bubble growth process in Cu at 300 K. He atom is added to the central bubble one by one with 60 ps interval. (**a**–**c**) correspond to 48, 49, and 136 He atoms in central bubble, respectively. Color of atoms and dislocation type by Burgers vector are as indicated, and Cu atom size is reduced to improve visualization.

**Figure 2:**
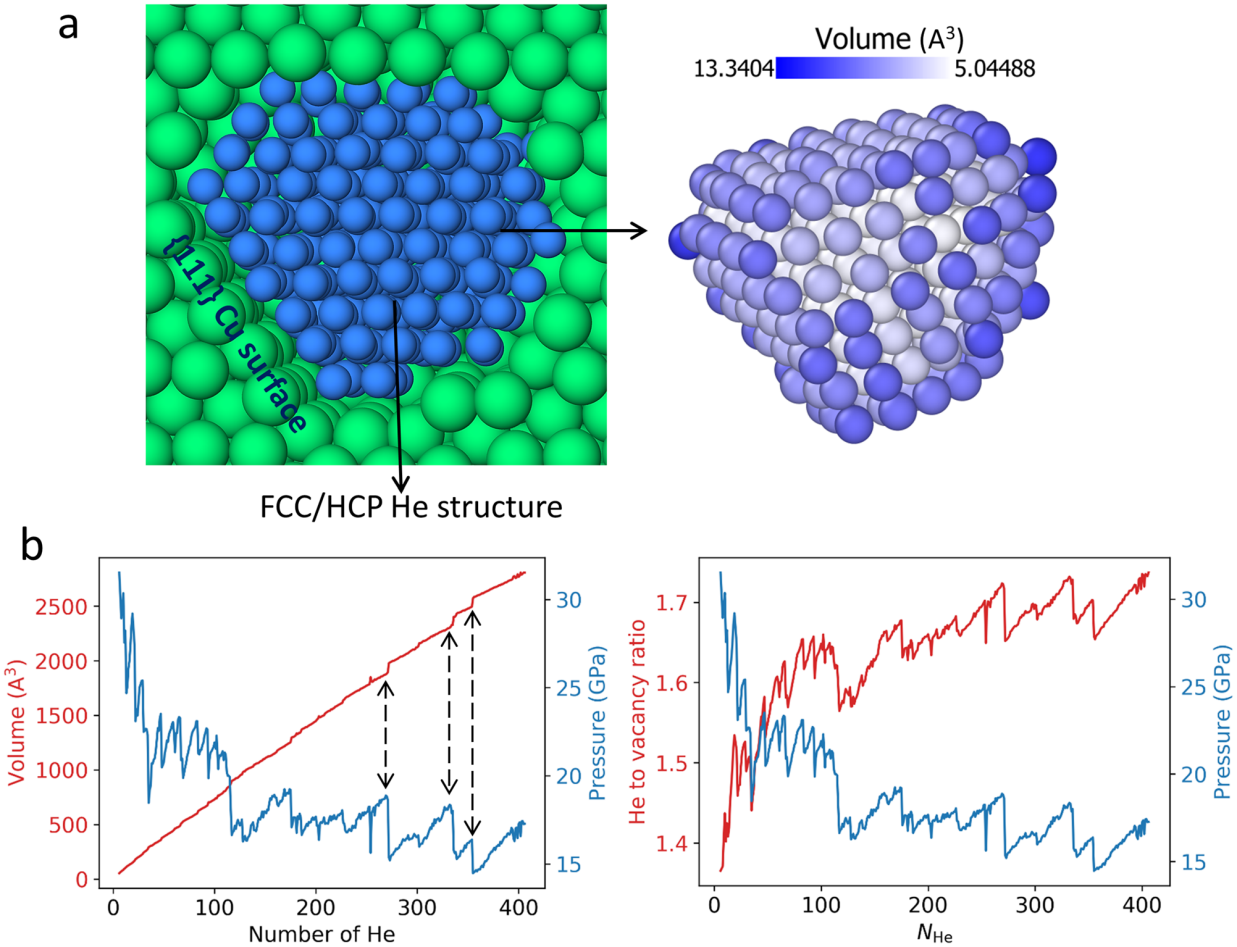
(**a**) shows the He bubble (266 He atoms) embedded in Cu matrix (blue: He, green: Cu). The bubble exhibits FCC structure, while HCP structure also occurs during the growth process. The Cu lattice exposes $\{111\}$ surface to the bubble. The bubble is in an imperfect octahedron, with atoms colored by the Voronoi volume. (**b**) depicts the time-averaged bubble volume, He to vacancy ratio, and pressure with respect to $N_{\mathrm {He}}$. The abrupt changes in volume and pressure are correlated and highlighted by vertical arrows.

The bubble growth process is simulated by successive He insertion in the central bubble with a fixed duration of dynamics in between to allow for system response (see “Methods” for details). This is a reasonable simplification of scenarios where He accumulation is achieved via efficient He interstitial diffusion. The temperature and bubble growth rate effects are examined via simulations at 300 K, 600 K, and 800 K, and He loading interval of 60 ps and 200 ps. Without loss of generality, the following results are reported based on the condition of 300 K and 60 ps interval if not otherwise specified. Figure 1 displays the highlight of this work which will be interpreted in depth. It visualizes the bubble growth process with selective atomic configurations, where the bubble contains 48, 49, and 136 He atoms, respectively (see supplementary video for the whole process). As He increases, Cu atoms are pushed out near the bubble surface (similar to the W-He system where self-interstitial atoms (SIAs) generated stay close to the bubble^17^) and develop a dislocation network around the bubble, mostly in Shockley partials. This is energetically more favorable than the emission of single interstitials as the binding energy between single interstitials is saved. Then these dislocations detach from the bubble after evolving into a DPDL. This is distinct from the previous work on prismatic loop punching with a single Burgers vector, e.g.,^6,11^. The DPDL is framed by $\frac{1}{6}\langle 112 \rangle$ dislocations, and linked by $\frac{1}{6}\langle 110 \rangle$ and $\frac{1}{3}\langle 001 \rangle$ type segments. The junctions obey the summation rule of Burgers vectors (see supplementary information 1 for detailed annotation). Although one may think that Frank loop may be more energetically favorable when loop is small and unfault to the perfect loop at large sizes^18^, Frank partial (${\mathbf {b}}=a/3\langle 111\rangle$) is actually not observed during the process. This is because the Frank partial is sessile, which can not release the local strain effectively; hence, the stress field from the over-pressurized bubble causes the dissociated loop energetically favorable. This loop can migrate rapidly in $\langle 110 \rangle$ direction (Fig. 1a,b), and then it is decorated and pinned by an accumulation of mobile He atoms from the bulk (Fig. 1b,c). As He continues to increase in the bubble, additional dislocation networks start to develop (Fig. 1c). Moreover, the bubble becomes faceted in a shape of an imperfect octahedron, built by close-packed $\{111\}$ Cu planes with lower surface energy^19^, and dislocation lines extend from the edges. This is consistent with the experimental work by Wei et al.^20^. Although it is intuitive to treat a bubble as spherical, the faceted bubble has long been reported^21,22^. To this extent, a pressurized bubble share the same characteristics with a void, with some facets lying on the close-packed planes to achieve a lower energy state with minimum energy surfaces. It is expected that this faceted feature is not a necessary condition for loop punching, but represents an energy favorable state (e.g., Wulff construction). There are at least two reasons: (1) it is known that punching is caused by the high bubble pressure; and (2) in the current study, the bubble becomes faceted as size increases, however, the loop punching occurs at the early stage where the faceting is still not obvious. From common neighbor analysis (CNA), He atoms in the bubble form FCC lattice shown in Fig. 2a (as also reported in^23^)). Hexagonal close-packed (HCP) stacking also emerges from bubble surface as the bubble size increases (check supplementary video for visualization). The FCC/HCP local structure grows with preferential $\{111\}$ surface orientation. Since He is known to have HCP as the most stable structure under high pressure^24^, the appearance of FCC stacking may relate to factors including the small bubble size, anisotropic matrix constraint, and capability of interatomic potential. The lift-out view (Fig. 2a right) depicts the bubble He atoms, colored by Voronoi volume. It demonstrates that the bubble interior is compact with individual atomic volume around 5.0 Å$^3$, while the surface He atoms occupy a much larger volume, especially corner atoms. Such volume distribution is facilitated by the surface diffusion of Cu atoms along the interface, which also assists the appearance of faceted configuration as He atoms are consecutively introduced into the cavity. In fact, surface diffusion along bubble-metal interface can lead to bubble migration^16^, although this process has not been observed in the current simulations due to limited timescale.

Bubble volume and pressure are quantified with respect to the constituent He atoms $N_{\mathrm {He}}$ (Fig. 2b). The values are obtained by averaging over 50 ps thermal equilibrium simulations of each atomic configuration to reduce fluctuation. The volume is calculated by summing the constituent He Voronoi volumes, and pressure is calculated by averaging the stress tensor of each He atom in the bubble, where computing stress tensor is detailed in LAMMPS^25^. The bubble volume scales almost linearly with $N_{\mathrm {He}}$. By contrast, the pressure decreases intermittently with increasing He atoms, with significant fluctuations. It is worth noting that pressure for a small size He cluster is not well-defined due to large per-atom fluctuations. Generally, the pressure and volume are well-correlated: the kinks in volume curve indicate pressure jumps (highlighted in Fig. 2b). The formation of vacancy can be inferred from the expansion of the bubble volume. Then He to vacancy ratio can be calculated by assuming constant vacancy volume equal to $a^3$/4, where a is the lattice constant (3.635 A at 300 K). Here, we neglect deformation of the lattice, especially near the interface. Figure 2b shows that He to vacancy ratio gradually increases to a plateau (1.6–1.7) as $N_{\mathrm {He}}$ increases. Such an increase is because as $N_{\mathrm {He}}$ increases, the gap between the bubble and matrix also increases, which causes more volume not treated as part of the bubble in the Voronoi tessellation scheme. Notice that the subtle features of He to vacancy ratio profile match the change in pressure extremely well: pressure increases with He to vacancy ratio before each punching at which point pressure drops. The high He to vacancy ratio is the key to enabling this punching mechanism, much higher than the previous studies of the optimal (or most stable) ratio in tungsten (1.0)^26^, iron (1.3)^27^ and vanadium (0.9)^28^.

**Figure 3:**
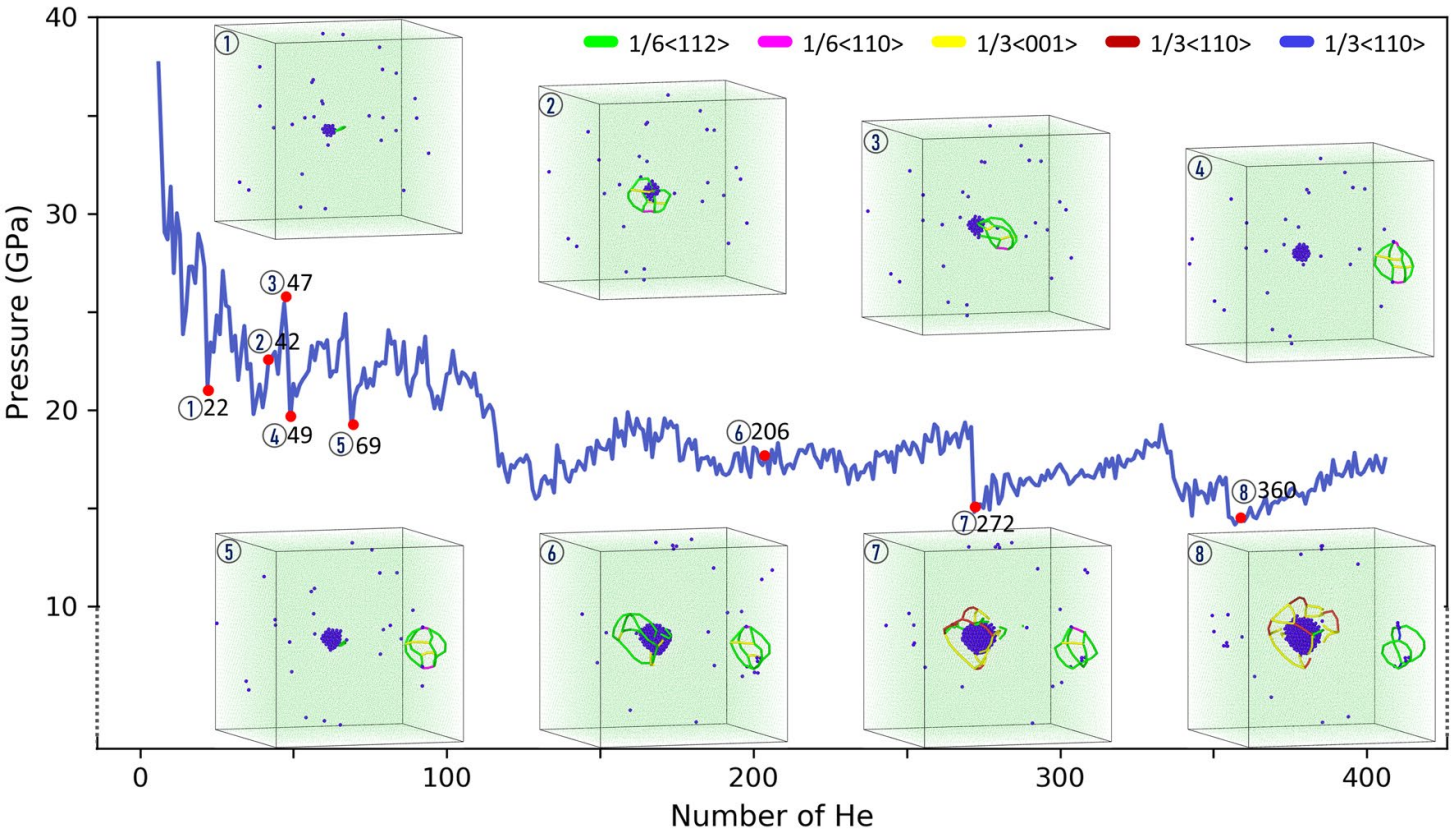
Instantaneous bubble pressure versus $N_{\mathrm {He}}$, marked with selective atomic and dislocation configurations. $N_{\mathrm {He}}$ is indicated beside the red points. Dislocations are colored by Burgers vector type.

**Figure 4:**
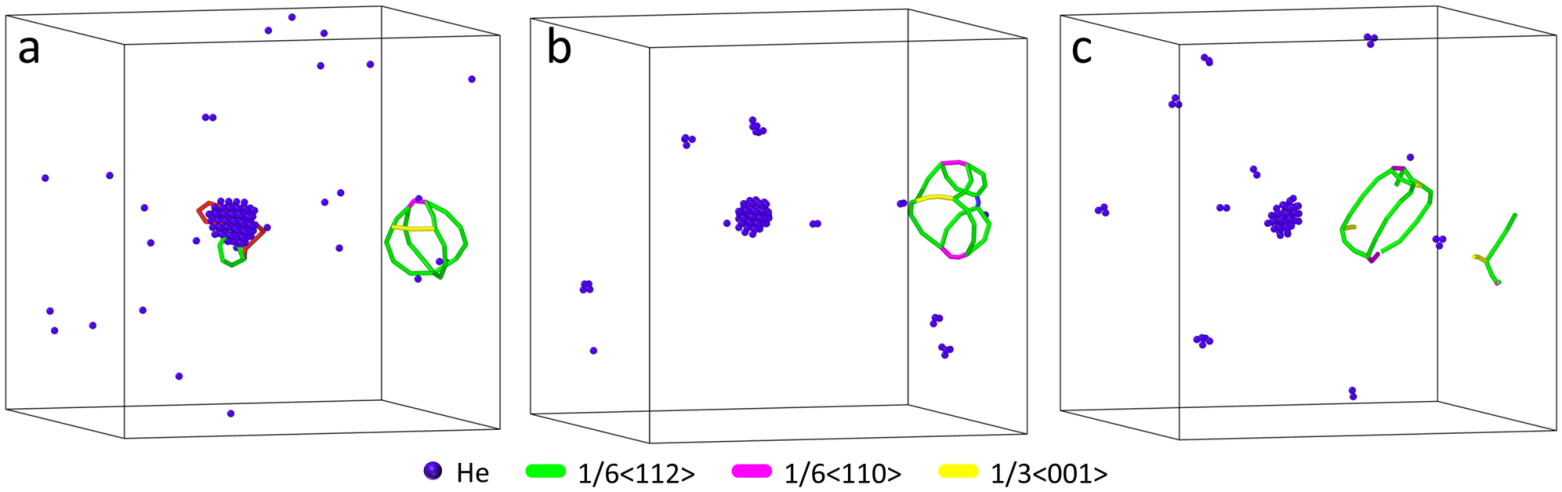
DPDL punching at 300 K (**a**), 600 K (**b**) and 800 K (**c**). Color of atoms and dislocation type by Burgers vector are as indicated; Cu atoms are removed to improve visualization.

The pressure, sensitive to local structural change, is an effective indicator of bubble-matrix evolution. Figure 3 plots the instantaneous (no averaging) bubble pressure marked with corresponding atomic configurations and dislocations. Upon nucleating dislocations, pressure is drastically reduced, e.g., He 22, 69, and 272 noted in the figure. As expected, the emission of DPDL releases bubble pressure and volume expansion occurs right at the point of loop punching; however, as it migrates away from the bubble (i.e., during the traveling stage), there is no obvious increase of volume, contrary to the analytical analysis of Ni punching a prismatic loop by Wolfer^11^. As bubble size increases, additional dislocations are nucleated at the bubble surface. Instead of punching another DPDL, they form large Burgers vector (${\mathbf {b}} = \frac{1}{3}\langle 110 \rangle$ or $\frac{1}{3}\langle 100 \rangle$) dislocations, which also leads to pressure release, e.g., He 272 and 360. These dislocations concentrate high strain energy ($\propto {\mathbf {b}}^2$) imparted by the growing bubble. Combining with Fig. 2b, such process also leads to volume jump, indicating a dynamic competition between lattice strain energy and bubble free energy. Due to the abrupt dislocation nucleation causing significant variations in pressure, the evaluation of equation of state (EOS) was not performed. The temperature effect is examined via the bubble growth process at multiple temperatures (300 K, 600 K, and 800 K($\sim 0.6\, T_m$)). Figure 4 visualizes the punched DPDL in simulations at the corresponding temperatures. Notably, the critical size of the bubble at which the initial DPDL detaches from the bubble decreases as the temperature increases (Table 1). This is presumably due to the increased bubble pressure with increasing temperature and increased probability of overcoming the activation barrier for loop emission. Similar temperature dependence was reported in the Fe–He system regarding punching an interstitial^29^. On the other hand, the DPDL size in terms of the number of the constituent SIAs is examined (Table 1). There is no clear correlation with temperature, due to two competing processes: (1) a higher temperature leads to punching at a smaller bubble size, which tends to punch out a smaller DPDL; (2) enhanced SIA diffusivity and clustering around the bubble tends to increase the DPDL size. Overall, the loop dimension is still on the same order as the bubble. The assumption used in literature (e.g,^1,30^) that loop size is around (or less than) bubble size in the theoretical treatment of loop punching criteria can still be reasonable, but it is worth noting that, depending on the loop configuration, the dimension of the loop can be larger than the bubble. The impact of growth rate is examined by increasing the interval between consecutive He loading. Here two variations are considered: $\Delta t = 60$ ps and $\Delta t = 200$ ps. Note that due to the limited timescale of MD simulations, both intervals still correspond to a very high He loading rate. The statistical variations in quantifying the critical bubble size overshadow any definitive judgment on the impact (Table 1). However, if given more realistic He loading rates and since a high interval provides extra time for loop formation and emission given the activation energy barriers, it is expected that the critical size of the bubble for DPDL punching should decrease (same effect as to increasing temperature).

**Table 1:** The critical size of He cluster to cause an initial DPDL emission, and the DPDL size in terms of its constituent SIA number.

Temperature (K)	300		600		800	
$\Delta t$ (ps)	60	200	60	200	60	200
$N_{\mathrm {He}}$	49	57 ± 4	46	45 ± 8	39	43 ± 10
DPDL size	27	–	34	–	32	–

$\Delta t=60$ ps columns correspond to the same simulations for Fig. 4. $\Delta t= 200$ ps columns use the average from $\sim$ 10 simulations with different random seeds.

**Figure 5:**
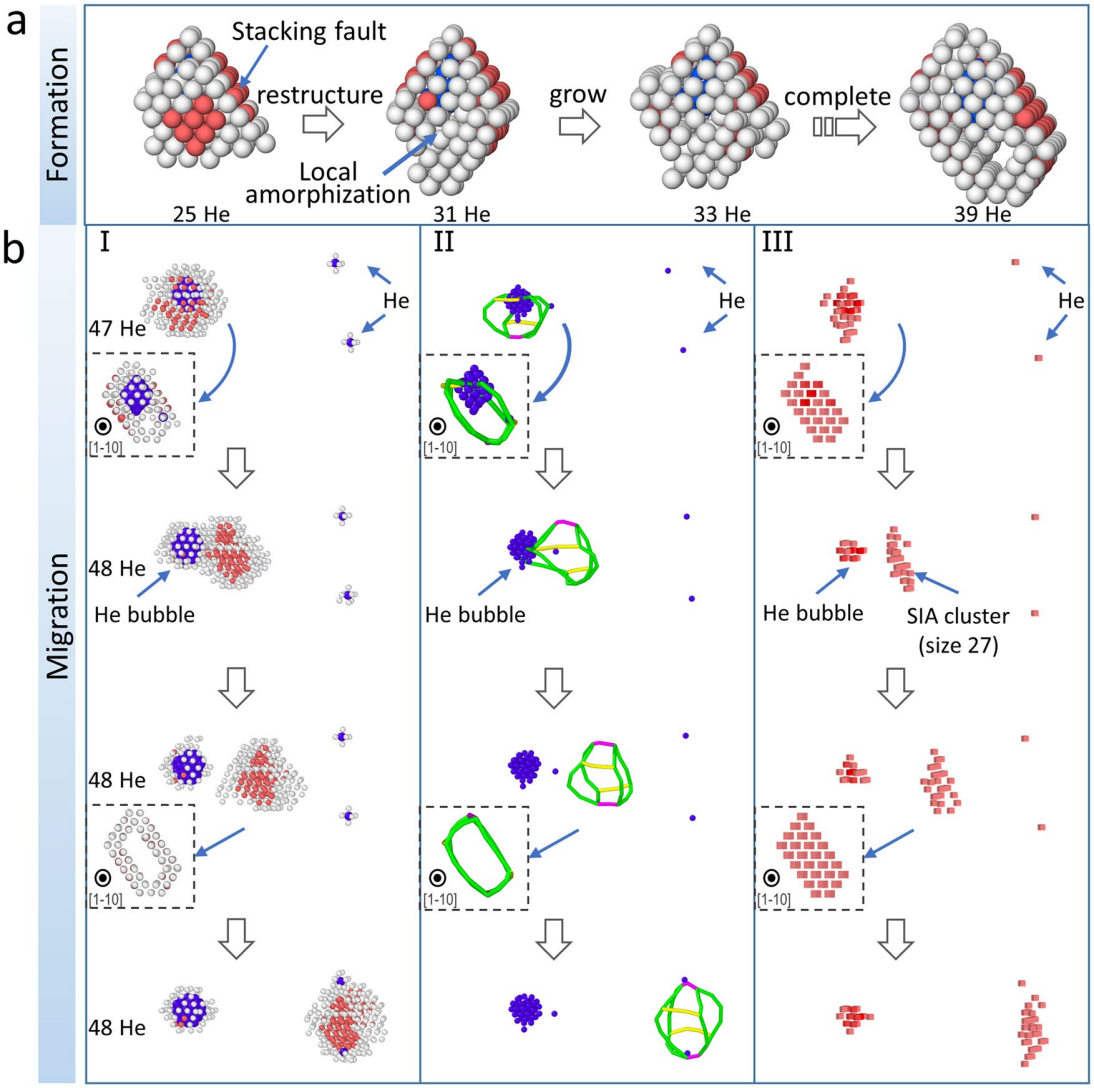
(**a**) shows DPDL formation process based on CNA with increasing He atoms in the bubble, where red, gray, and blue balls indicate HCP Cu, non-FCC/HCP Cu, and He atoms, respectively. The rest atoms are removed to improve visualization. The DPDL is formed by gradually growing stacking faults around the bubble in parallelepiped shape. (**b**) shows the DPDL migration process from concurrent CNA (I), dislocation analysis (II, legend consistent with Fig. 1), and defect analysis (III, red cubes indicating interstitials), respectively. The insets demonstrate views from [−110] direction. $N_{\mathrm {He}}$ is annotated.

The formation and migration of DPDL are illustrated in Fig. 5. At the early stage, bubble growth gradually punches out SIAs which tend to form stacking faults. As more SIAs are created, the stacking faults grow and organize into a parallelepiped shape with stacking faults on each peripheral four faces at two sets of parallel $\{111\}$ planes, encompassing the bubble (Fig. 5a). Upon completion of the DPDL, a burst-like emission driving a pressure release is shown in Fig. 5b from three perspectives, i.e., common neighbor, dislocation, and defect analysis, respectively. It demonstrates that, after one more He is added to the bubble, debris dislocation segments are pinched from the bubble surface, and a complete DPDL is emitted in [1−10] direction. The migration is driven by stress, and proceeds progressively rather than synchronized (Fig. 5b). By defect analysis, the mobile DPDL contains 27 SIAs on multiple $\{110\}$ planes, with morphology changing during migration. Ultimately, the structure becomes immobile as it approaches the edge of simulation cell and two He interstitials diffuse from the bulk to reside on the dislocation segments. No further migration is observed at this temperature (300 K), but the partials are constantly oscillating, as additional long simulation ($\sim$ ns) is performed. Referring to Fig. 3$\textcircled{\,\,8}$, the stacking faults can revert back and forth to perfect dislocations ($1/2\langle 110 \rangle$), indicating competing strain energies. By increasing the temperature, more intense DPDL migration can be observed: the initial DPDL can interact with subsequent dislocation networks tied to the bubble, develop an enlarged DPDL, and then detach in a $\langle 110\rangle$ direction again (see supplementary information 1 for visual demonstration).

## Discussion

In contrast to voids (zero/low internal pressure) which mostly grow by absorbing mobile vacancies, coarsening, and coalescence (it should be noted that voids can also grow by dislocation emission under external stress such as shock loading^31,32^), bubble growth can be athermal in the form of dislocation/interstitial punching as the excess high bubble pressure pushes the surrounding matrix atoms^8,17,28,29,33–35^. It can happen under a high He production rate and/or low temperature^30,1^. Comparing the bubble growth process dominated by punching in various metals, it can be seen that a high bubble pressure ($\sim$ tens of GPa) is expected, much higher than the typical yield stress of the matrix. The bubble volume then grows at the expense of interstitials punched out. The current study (i.e., high-pressure scenario) expands upon the atomistic details of the formation, configuration, and migration characteristics of dissociated dislocation loops during the athermal bubble growth in Cu, which have not been resolved before. The critical pressure for punching can be analyzed from the energetics point of view: the prismatic loop will be generated if the formation energy of the loop is smaller than the work done to increase the bubble size. Based on continuum theory, Trinkaus^30^ arrived at the following criterion for loop punching,

1 $$\begin{aligned} p\ge (2\gamma _s+Gb)/r_L \end{aligned}$$

where *p* is the bubble pressure, $\gamma _s$ is the Cu surface energy (approximated as 1.952 J/m$^2$ for (111) surface^36^), *G* is the shear modulus ($G_{\text {Cu}}$ = 45 GPa), *b* is the magnitude of the Burgers vector (2.54 A), and $r_L$ is the loop radius. Assuming the loop radius equal to that of the bubble ($r_B\sim$ 1.0 nm), then the required pressure for loop punching would be at least 15.3 GPa. Note that the real necessary pressure is probably larger than this value, due to the inherent inaccuracy of the theory in describing the energies and neglect of the activation energy barrier for loop punching. This estimation, although smaller, agrees reasonably with our calculations (Fig. 3). Comparing the first and second term (i.e., 2$\gamma _s$ vs. *Gb*), *Gb* contributes much more to the summation. It means that, for a metal with a high shear modulus, the critical pressure is expected to be high; this can be quantitatively reflected from the study of the W-He system^34^, which reports a much higher pressure to initiate loop punching than the value in Cu (note: $G_{\text {W}}$ = 161 GPa and $G_{\text {Cu}}$ = 45 GPa).

Holistically, this DPDL, in an approximate parallelepiped shape, can be condensed into a perfect dislocation loop with Burgers vector $\frac{1}{2}\langle 110 \rangle$ from two extra $\{110\}$ planes. In Fig. 5b III, there are a total of 27 SIAs, which can geometrically span the two planes across around 125 Å$^2$ (estimated based on lattice constant $a =$ 3.597 Å); it agrees with the direct examination of the cross-sectional area of the loop from its configuration (see supplementary information 1 for details). From Fig. 5a, the four faces are on two sets of parallel $\{111\}$ planes are stacking fault ribbons bounded by Shockley partials. The ribbon width (*d*) can be estimated through a force balance between the repulsive force between the partial dislocations and attractive force due to the stacking fault energy ($\gamma$)^18^, i.e., $d \approx G\mathbf {|b|}^2/(4\pi \gamma )$, where $|{\mathbf {b}}|=a/\sqrt{6}$. Plugging in the numbers for Cu, with $G=45$ GPa, $\gamma = 45~ \mathrm {mJ/m^2}$^37^, and $a = 3.597 ~\mathrm{A}$, we can obtain $d\approx$ 17 Å. This value agrees with direct examination (Figs. 1, 4), giving an estimated volume around 2.2 $\mathrm {nm}^3$ to accommodate these SIAs, which can be technically captured in experiments with transmission electron microscopy (see supplementary information 1 for possible appearance). From the current study, the dimension of DPDL can be larger than that of the bubble, contrary to both the finding in BCC metals where the size of punched loop is about the same as the bubble^6,9^ and the assumption used in the previous theoretical estimation of punching criterion^10,30^. This results from the interstitial distribution around the bubble and energetically favorable loop configuration in the presence of over-pressurized bubble. As the loop configuration has been determined, the largest loop can be estimated by assuming that the SIAs created during the bubble growth process all cluster into the DPDL. In this case, the dimension of DPDL $r_L$ correlates with $r_B$ such that $r_L\sim 1.89{\sqrt{r_B^3/a}}$ (assuming spherical bubble and circular loop). This can be achieved with elevated temperatures due to enhanced diffusion and DPDL formation (see supplementary information 1 for demonstration at 800 K). On the other hand, from Eq. (1), it suggests that emission of large loops is more likely than small loops. However, the energy barrier to form a loop is expected to increase with the loop size, which kinetically limits the size of emitted loops.

Migration of DPDL can be seen as gliding of leading and trailing Shockley partials, and the migration itself is mechanically actuated by the strong repulsion between the over-pressurized bubble and the interstitial loop. Therefore, it is reasonable to extend that this mechanism should also be active in other low stacking fault energy metals such as Ag and Au, since DPDL represents an energetically favorable form to effectively release local strain imposed by highly pressurized bubbles. As the bubble grows, the bubble becomes faceted, and Cu matrix exposes $\{111\}$ interfaces (Fig. 2a and experimental validation in^20^). Thus, the impingement of high pressure mimics the typical nanoindentation with $\{111\}$ surface orientations of metal samples. Indeed, similar DPDL has been found in multiple FCC metals under nanoindentation including both low and high stacking fault energy metals such as Ni^38^, Al^39^, and Au^40^. For example, Lee et al.^40^ reported a burst-like emission of these loops nucleated from the contact surface in Au using in-situ TEM. Hence, the initiation of plasticity by nucleating dislocations due to high pressure and punching out prismatic loop exhibits consistency. Therefore, it is expected that DPDL punching during the athermal bubble growth can occur in general FCC elemental metals (except that with extremely high stacking fault energy which would prevent any dissociation of the full dislocation). However, the difference of stress field from indenter and bubble results in a different DPDL formation mechanism in the latter: interstitials are generated surrounding the bubble, which leads to the nucleated loop embryo encompassing the bubble (i.e., $r_L \gtrsim r_B$). In the case of very large bubbles (hence low pressure), local high stress can punch out interstitials, leading to the formation of dislocations and localized dislocation loop close to the bubble (i.e., $r_L < r_B$, see supplementary information 1 for possible appearance from heat treatment).

Bubble growth and dislocation loop, which constitute the main cause of mechanical degradation such as hardening and embrittlement^1^, are closely related: athermal bubble growth can be facilitated by dislocation loop punching, and dislocations can be nucleation sites for nascent bubbles^12,41^. This new growth mechanism found in Cu expands upon the previous mechanistic understanding of the bubble growth process. Here, the DPDL induced by bubble growth will play an important role in the microstructure evolution. In the simulations, the sparse He atoms in the system tend to accumulate at the DPDL, especially the junctions and segments with a large Burgers vector. With continuing He implantation and assistance of dislocation pip diffusion, bubble nucleation and growth is bound to occur at DPDLs. Such phenomenon can lead to enhanced bubble population than the prediction based on conventional rate theory models^1^. In addition, the DPDL moves along $\langle 110\rangle$ directions, and becomes stationary at the simulation cell edge where the forces from the over-pressurized bubble cancel out due to the periodic boundary condition (He accumulation at DPDL is supposed to provide additional pinning as He cluster becomes large^42^). This observation has important implications for the reported He bubble superlattice in Cu with a FCC symmetry^43^, which means a preferential growth of bubble arrays in $\langle 110\rangle$ directions. Therefore, such immobilized DPDLs can be a precursor in forming such a self-organized superstructure of bubbles by providing an “anchor” for new bubbles. By extension, this understanding may also apply to other FCC metals aforementioned, which similarly form gas bubble superlattice under irradiation^44^.

## Conclusion

In this study, by gradually increasing He atoms in the bubble, we unveil a unique mechanism in He bubble growth in Cu, which arguably applies to general FCC elemental metals. Initially, the high pressure of a small bubble pushes out Cu interstitials which then forms a dislocation network near the bubble surface. This network finally transform into a DPDL consisting of stacking faults on multiple $\langle 111 \rangle$ planes bordered by Shockley partials, in a shape of parallelepiped. This loop is emitted in $\langle 110 \rangle$ direction, becomes stationary as it approaches the edge of the simulation cell, and can capture diffusive He atoms to potentially become a new bubble nucleation site. For the He bubble, the pressure is intermittently reduced while punching more Cu interstitials to nucleate new dislocations, and the critical size of the bubble for DPDL punching decreases with increasing temperature. The bubble itself exhibits a compact lattice structure in FCC/HCP, and the Cu side energetically favors $\{111\}$ surface orientations. These atomistic findings complement the existing experimental work, and underpin the unique fundamental processes from athermal bubble growth in FCC metals.

## Methods

MD simulations based on the LAMMPS^25^ package are used to examine He bubble growth process in Cu. The atomic interactions are described by an EAM potential^45^, which has shown a high predictability in He impurity energies in FCC Cu. An initial structure containing $12\times 12\times 12$ FCC unit cells of Cu is constructed, and adequately relaxed at 300 K under zero pressure for 100 ps, with periodic boundary conditions applied in all axial directions. Then a He cluster of size 6 is generated in the center of simulation cell and 30 additional He atoms are scattered across the cell. These scattering He atoms to model realistic conditions can unveil phenomena beyond single bubble simulations. Then, after 60 ps of canonical simulation, He atom is inserted one by one into the central He cluster. After each insertion, there is 60 ps additional run to allow the system to respond upon an increasing pressure of the central bubble. Nose-Hoover thermostat^46,47^ is used to maintain the system temperature at 300 K. The evolving atomic configurations are recorded for structural analysis and visualization, accomplished using the OVITO package^48^. In addition, simulations with higher temperatures (600 and 800 K) and a prolonged interval (200 ps) between He loading are also performed to explore the temperature and growth rate effect on the punching process. Quantification of the bubble pressure and volume is based on atom stress tensor and Voronoi analysis, respectively.

## Supplementary Information

Supplementary Information 1.

Supplementary Information 2.

## Data Availability

The datasets that support the findings of this study and relevant scripts for replication are available in the Zenodo repository (https://doi.org/10.5281/zenodo.3970979).
