# Supplementary material for: Dissociated prismatic loop punching by bubble growth in FCC metals
Source: Sci Rep. 2021 Jun 18;11:12839. doi: 10.1038/s41598-021-92219-7 (PMC8213783; doi:10.1038/s41598-021-92219-7)
Supplement: Supplementary file 1 — Supplementary Information 1. [file 41598_2021_92219_MOESM1_ESM.pdf]

Supplementary information to manuscript:

Dissociated prismatic loop punching by bubble growth in FCC metals

Miaomiao Jin,[*a] Yipeng Gao,[b] Yongfeng Zhang,[c] Chao Jiang,[b] and Jian Gan[b]
[a] Department of Nuclear Engineering, Penn State University, 205 Hallowell Bldg, University Park, PA 16802, USA
[b]Idaho National Laboratory, 2525 Fremont Ave, Idaho Falls, ID 83402, USA
[c]Department of Engineering Physics, University of Wisconsin, 1500 Engineering Drive, Madison, WI 53706, USA
[*]Corresponding author: mmjin@psu.edu

1. General dislocation punching

In the scenario of high helium production rate and low vacancy supply, bubbles grow by the athermal

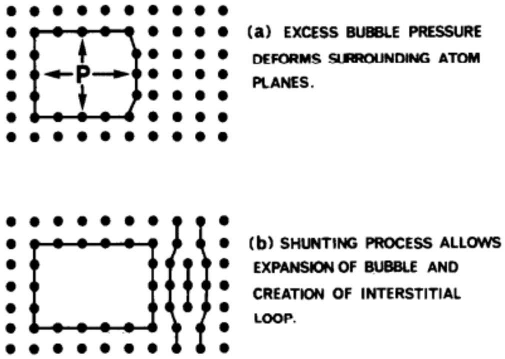

Figure 1. Mechanism of bubble growth by loop punching from reference [1] Fig. 3.

processes including interstitial and dislocation punching, which are driven by the high pressure. Figure 1 provides a schematic demonstration of the loop punching, which indicates the bubble volume expansion is at the expense of expulsion of matrix atoms, leading to the formation of dislocation loop. Trinkaus et al. [2] analyzed the loop formation criteria by comparing the free energy change before and after the loop formation, and found out the critical pressure for this process depends on the mechanical properties of the matrix (poission ratio and shear modulus), and the bubble and loop sizes. However, the detailed characteristic of punching during bubble growth is materials dependent. In the current study (i.e., high-pressure scenario), we expand the atomistic details of the formation, configuration, and migration characteristics of the dislocation loop in Cu, which have not been resolved before.

2. Dislocation analysis of dissociated prismatic dislocation loop (DPDL)

The dislocation types by Burgers vector are annotated in Figure 1a for the dissociated loop and schematically illustrated in 1b, where the colored faces are stacking faults bordered by 1/6<112> Shockley partials. This dissociation is energetically favorable than the perfect loop 1/2<110>.

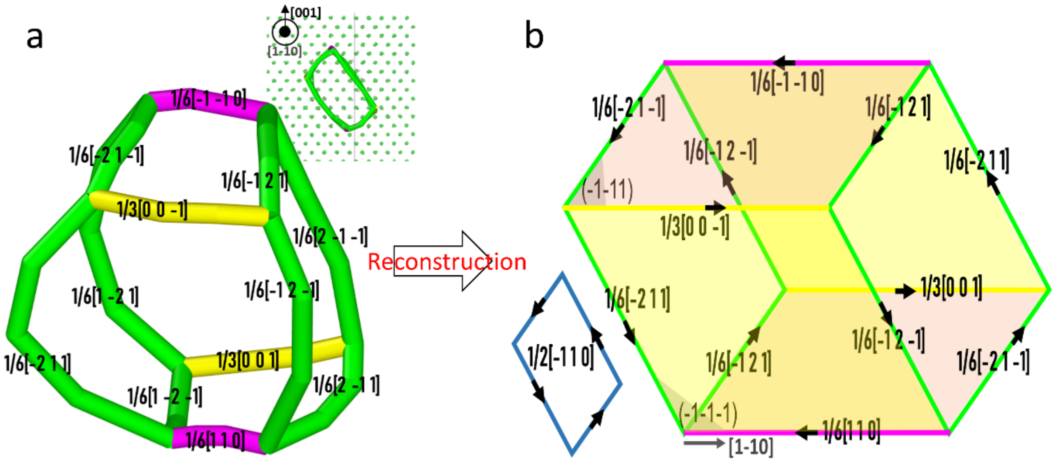

Figure 1. (a) Dislocations distinguished by Burgers vector for each segment of the dissociated prismatic dislocation loop. (b) Reconstruction of the loop where the four faces indicate stacking faults. The dissociated loop is an energetic favorable form than the perfect dislocation loop (1/2<110>).

3. Temperature effect

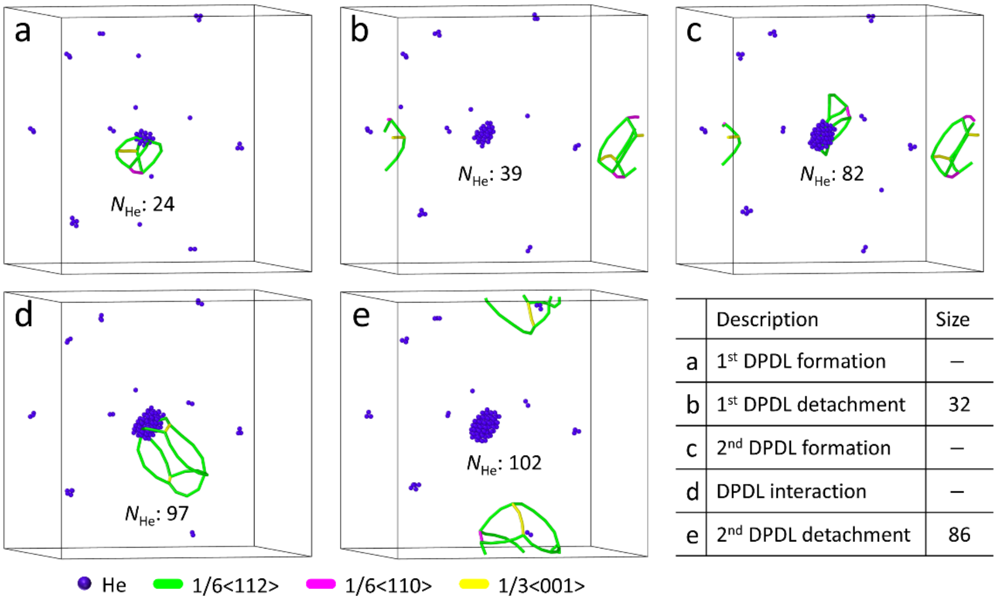

	Description	Size
a	1st DPDL formation	–
b	1st DPDL detachment	32
c	2nd DPDL formation	–
d	DPDL interaction	–
e	2nd DPDL detachment	86

Figure 2. Bubble growth and DPDL punching at 800 K. N_{He} denotes the number of He atoms in the bubble. “Size” in the table denotes the number of constituent SIAs in the DPDL. Color of atoms and dislocation type by Burgers vector are as indicated; Cu atoms are removed to improve visualization.

Figure 2 demonstrates the bubble growth process and the corresponding DPDL punching at 800 K. Unique features are revealed as the temperature is increased. The accelerated diffusion of SIA and higher bubble internal pressure cause efficient emission of DPDL (the critical size of the bubble to punch an initial DPDL decreases with increasing temperature). Moreover, the interaction between 1st and 2nd DPDL leads to a DPDL at a much large size (Figure 2e), containing 86 Cu SIAs.

4. Loop size estimation

The loop area can be estimated based on the number of interstitial atoms (N_i) constituting the loop. Approximating the interstitials to span two {110} extra planes (Figure 3a), as the per-atom area $A = \frac{\sqrt{2}}{2}a^2$ (Figure 3b), where a is lattice constant 3.597 A, therefore, the loop area $A_{\text{loop}} = \frac{N_i}{2}A = \frac{\sqrt{2}N_i}{4}a^2$

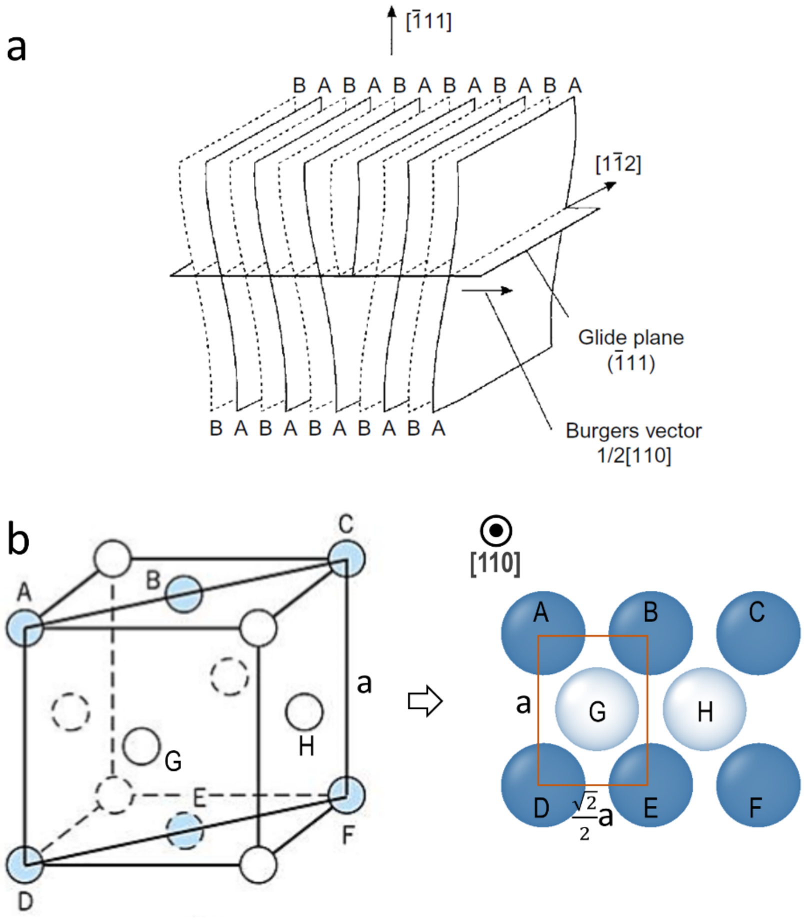

Figure 3. (a) Unit edge dislocation ½[110] in a face-centered cubic crystal, from [1]. (b) Unit cell and atoms from [110] direction (dark and light blue correspond to A and B layers in (a)).

The size of largest DPDL in the simulation cell can be estimated by considering that all interstitials punched out by the bubble cluster into the DPDL. In this case, we can obtain a rough correlation between loop radius r_L and bubble radius r_B as follows,

$$b\,\pi r_L^2 = \frac{4}{3}\pi r_B^3$$

where $b = \sqrt{2}/2\,a$

$$r_L = \frac{1.89}{\sqrt{a}} r_B^{\frac{3}{2}}$$

5. Experimental evidence of DPDL punching

Wei et al. [7] performed He irradiation experiment with Cu and use TEM to characterize the microstructure at stages of heat treatment, specifically on the evolution of bubbles. Based on their observations, Figure 4 extracts point of interest relevant to current study where the contrast change in the diffraction pattern indicates a potential DPDL punching process. Note that the bubble size is much larger than that in the simulations and the supposed loop emission occurs at the side of the bubble. This process ought to be facilitated by the heat treatment noted in the figure, so that the interstitials punched out due to bubble growth can form DPDL aside the bubble via surface diffusion. Additional characterization is desired to confirm the DPDL structure since this phenomenon was not specifically focused and examined in the original experiment.

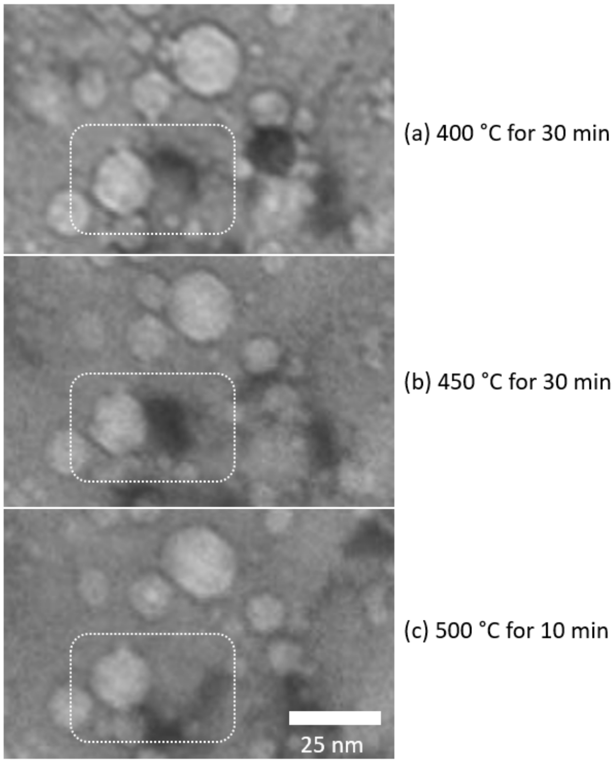

Figure 4. TEM diffraction contrast changes gradually in response to evolution of bubbles (cross-sectional TEM under-focused images) in He-irradiated Cu under a sequence of heat treatment as indicated, adapted from [2] with permission.

REFERENCE

1. Evans, J. H. "The role of implanted gas and lateral stress in blister formation mechanisms." *Journal of Nuclear Materials* 76 (1978): 228-234.
2. Trinkaus, H., and W. G. Wolfer. "Conditions for dislocation loop punching by helium bubbles." *Journal of Nuclear Materials* 122, no. 1-3 (1984): 552-557.
3. Hull, Derek, and David J. Bacon. *Introduction to dislocations*. Butterworth-Heinemann, 2001.
4. Wei, Qiangmin, et al. "The shape of bubbles in He-implanted Cu and Au." *Scripta Materialia* 63.4 (2010): 430-433.
